# Student Presenteeism in Digital Times—A Mixed Methods Approach

**DOI:** 10.3390/ijerph192416982

**Published:** 2022-12-17

**Authors:** Rebecca Komp, Simone Kauffeld, Patrizia Ianiro-Dahm

**Affiliations:** 1 Department of Management Sciences, Bonn-Rhein-Sieg University of Applied Sciences, 53359 Rheinbach, Germany; 2 Department of Industrial/Organizational and Social Psychology, Institute of Psychology, Technical University Braunschweig, 38106 Braunschweig, Germany

**Keywords:** presenteeism, students, health, ability to study, digital learning, health promotion, mixed methods study

## Abstract

In young adulthood, important foundations are laid for health later in life. Hence, more attention should be paid to the health measures concerning students. A research field that is relevant to health but hitherto somewhat neglected in the student context is the phenomenon of presenteeism. Presenteeism refers to working despite illness and is associated with negative health and work-related effects. The study attempts to bridge the research gap regarding students and examines the effects of and reasons for this behavior. The consequences of digital learning on presenteeism behavior are moreover considered. A student survey (*N* = 1036) and qualitative interviews (*N* = 11) were conducted. The results of the quantitative study show significant negative relationships between presenteeism and health status, well-being, and ability to study. An increased experience of stress and a low level of detachment as characteristics of digital learning also show significant relationships with presenteeism. The qualitative interviews highlighted the aspect of not wanting to miss anything as the most important reason for presenteeism. The results provide useful insights for developing countermeasures to be easily integrated into university life, such as establishing fixed learning partners or the use of additional digital learning material.

## 1. Introduction

Students have only been a target group for preventive and health promotion measures for a few years. However, there are some aspects that speak strongly in favor of focusing on students and strengthening their health awareness as follows: The first semesters in particular are characterized by numerous changes (e.g., leaving home, and new social contacts) [1]. These can lead to insecurities as well as stress and, consequently, impaired health and more risky health behaviors (e.g., less physical activity, more alcohol) [1,2]. This is particularly problematic because the crucial foundations for health in later life are laid in young adulthood. Engaging in risky health behavior as a young adult can have negative consequences for health in the long term. The university offers a good opportunity to implement disease prevention and health promotion measures before entering working life and thus strengthen health-promoting behavior [1]. It represents a setting where students spend a lot of time. According to the *Ottawa Charter* [3], health is created in exactly such places. The *Okanagan Charter* established the principles of a “*Health Promoting University*”. According to these, health should be integrated into university culture, processes, and policies in order to enhance health and well-being [4]. This is also of interest to employers, as students are the employees of tomorrow and a healthy lifestyle is needed to meet the demands of working life [1].

This article focuses on a field of research that is highly relevant to health but has been rather neglected in the student context, namely, the phenomenon of presenteeism. It refers to the behavior of working despite illness. Ruhle et al. [5] for example, define presenteeism in general terms as the “behavior of working in the state of ill-health”. The following various reasons may lead to presenteeism: acute or chronic diseases, contagious or non-contagious diseases. The consequences are different in each case. Especially in the case of chronic diseases (e.g., depression), presenteeism can have positive consequences. Ruhle et al. [5] have summarized preliminary studies showing the following positive effects: work is meaningful and can help fulfill basic psychological needs. There is also a distraction from the health impairments and isolation is avoided. However, in most cases, the negative consequences of presenteeism are of greater relevance. The negative effects on general health, well-being, and work ability [6,7] have been identified (see Section 2.1), which confirms the relevance of this topic.

Presenteeism in the workplace has been widely studied but not only are employees affected by presenteeism. Studies have shown that the time required for an average course of study is roughly the same as in working life and that the workload can therefore be considered similar [8]. In addition, many students also have a part-time job (on average, around 60% of students work during the lecture period [9]). This suggests that presenteeism also plays a role in the student context. The initial studies on the prevalence of presenteeism have been presented in recent years. These have revealed that two-thirds of students had engaged in presenteeism on at least one day in the previous year (average of 6.6 days) [10], up to two-thirds related to the last semester (average of 4.8 days) [11]. Presenteeism thus also seems to occur among students. This is problematic for the following reasons: students develop various health-related behaviors during their studies and maintain routines. If negative behaviors are exhibited here, they will affect behavior later in life. It can be expected that those engaging in presenteeism during their studies will likely persist in this behavior in working life. In addition, students will have poorer health as employees later in life if they have frequently exhibited presenteeism during their studies.

For these reasons, the study focuses on presenteeism among students and attempts to bridge the existing research gap regarding this target group. As described above, there are only a few studies with students as a target group. There is a lack of detailed findings on the effects of and reasons for presenteeism. However, the examination of this phenomenon is particularly relevant for this target group as the foundations for health in later life are laid in young adulthood.

Therefore, the first step is to quantitatively investigate whether presenteeism—similar to that among employees—is also associated with a poorer state of health, impaired well-being, and an impaired ability to study among students. The effects of the sudden increase in digital learning due to the Corona pandemic on the presenteeism behavior of students are also of interest here. A qualitative study will be conducted to determine the reasons for presenteeism, which is important for deriving needs-oriented measures.

## 2. Quantitative Survey

### 2.1. Theoretical Background and Hypotheses

Due to the prevalence of presenteeism among students, it is important to examine whether the negative health and work-related effects demonstrated in the work context also apply to students. It has been shown that employees who frequently go to work despite being ill have poorer long-term general health [7], more physical complaints [7,12], and poorer well-being [7]. The probability of future absences due to illness increases [7,13]. Furthermore, presenteeism leads to an impairment of the ability to work [6,7] and to productivity losses, which causes high costs [14]. For the target group of students, Töpritz et al. [15] provided initial evidence and showed that presenteeism is associated with poorer general health, health complaints, and burnout. However, presenteeism was understood there as impaired performance due to health problems [15]. Beyond this “economic” perspective, individuals and their health should be brought to the fore, as presenteeism encompasses more than just impaired performance due to health problems. As described, relationships with the general state of health, well-being, and the ability to work are known from the work context [6,7]. It should be examined whether these relationships can be transferred to students. It is assumed that *there is a negative relationship between engaging in presenteeism and*…

**H** **1a.** *… the general state of health.*

**H** **1b.** *…well-being.*

**H** **1c.** *… the ability to study.*

The question moreover arises as to whether the effects of the changed learning and study conditions due to the Corona pandemic are also related to presenteeism. The exact situation at the university investigated here at the time of data collection is described in the Methods section. During the Corona pandemic, teaching at universities was converted to digital formats, causing enormous challenges [16]. The effects of the change in learning are described below and the connection with presenteeism is derived on this basis as follows:

Students report a heavier workload in times of online teaching. In an international study of more than 30,000 students, almost half reported that their workload had increased (significantly). The reasons for this perception are, for example, the increased self-discipline and self-motivation required when studying alone at home [17]. The perception of a heavier workload results in a higher stress level [18]. In a study by van der Feltz-Cornelis et al. [19] as many as 70% of the students reported that their study-related stress had increased due to the Corona pandemic. The fact that a heavy workload [20,21] and the associated increase in stress [21] leads to more presenteeism has been proven several times in the work context. This relationship is also assumed to apply to students since they also want to avoid not doing their work when they experience a high level of stress and therefore persevere with their studies in spite of illness as follows:

**H** **2a.** *There is a positive relationship between the experience of stress and engaging in presenteeism.*

Another aspect characteristic of studying at home is the lack of psychological detachment. This refers to a physical and above all mental distancing from work. Detachment from work prevails when an individual does not work outside working hours and does not think about work [22]. This is difficult to achieve when the spatial boundaries between work and home are few or non-existent [23]. Students were confronted with this problem during the Corona pandemic as there was no other place, such as the library, in which to study. Thus, the same place that was used for studying during the day was often used for leisure and relaxation in the evening. Furthermore, some leisure activities contributing to detachment in normal times were not possible or only to a limited extent (e.g., cinema, sports club, etc.). This makes it more difficult to switch off from studies. In a meta-analysis, it was shown that detachment as a facet of recovery is positively related to the general state of health [24]. If it is not possible to switch off, negative health effects may ensue. According to the dual-path model [25], an impaired state of health renders subsequent presenteeism more likely. Therefore, the following hypothesis is postulated:

**H** **2b.** *There is a negative relationship between detachment from studies and engaging in presenteeism.*

The Corona pandemic and the resulting remote learning also brought about changes in terms of social support from lecturers and fellow students. If the courses are not held in person, communication also shifts to the digital world. This makes it more difficult for students to communicate with each other and to interact with the teachers [16]. We assume that this situation leads to increased presenteeism, as students with less support feel that they have more responsibility for themselves and therefore have to attend every course, even if they are ill. For employees, Lohaus and Habermann [21] demonstrated a negative relationship between presenteeism and organizational support. The following analogous assumption is made for students:

**H** **2c.** *There is a negative relationship between social support in university and engaging in presenteeism.*

### 2.2. Materials and Methods

#### 2.2.1. Procedure and Participants

A university-wide online student survey was conducted as part of the university’s student health management. The aim was to obtain a comprehensive picture of the state of health of all students so as to develop individual measures for students in the long term and also to improve the university-specific health management. The constructs listed here represent a subsection of the detailed survey. The survey period was October and November 2021.

It should be noted that the data were collected during the Corona pandemic. Although the university studied here had partially returned to face-to-face teaching at the time of the survey, the following after-effects of the preceding online semesters persisted: some of the courses were still offered online and the university’s facilities, such as the canteen and library, were still subject to restrictions. The pandemic was far from contained, and high numbers of cases continued to be recorded. Normal studying was far from being resumed.

A total of 1047 of the 9256 students contacted completed the questionnaire, giving a response rate of around 11%. Due to missing values, the analyses were finally based on fully completed questionnaires from 1036 students (61% female/39% male, predominantly 17–24 years old, see Table 1).

Note that the composition of the participants in the student survey corresponds to the general population of students at the university in essential characteristics (degree pursued, semester of study). This also largely applies to the participation from the various faculties (not shown here for reasons of anonymity). In terms of gender and age, more female and younger students participated than the general student population would suggest.

#### 2.2.2. Measures

The main construct, presenteeism, was assessed by four items from the German *Gesundheitsmonitor* (health monitor) [26]. An example item was as follows: “In the last 12 months, how often did you work on your studies (e.g., preparing for exams) even though you felt really sick?”. The response alternatives were 1 = “not once”, 2 = “once”, and 3 = “twice or more”. The original items were adapted for students by talking about studying instead of working. One of the five original items asked about leave days, which is not relevant in the student context and was therefore omitted.

The single item “How would you describe your health in general?” was used to measure the general state of health (1 = “poor” to 5 = “excellent”). The Study of Adult Health in Germany (DEGS) served as the basis for the formulation. The DEGS questionnaire is conducted within the health monitoring of the Robert Koch Institute and offers comparative data on various age groups [27].

Psychological well-being was assessed using the German version of the WHO-5 Well-Being Index (Cronbach’s alpha = 0.92) [28]. The scale consisted of five positively formulated items, such as “In the last two weeks I have been happy and in a good mood”. The associated response categories ranged from 1 = “at no time” to 6 = “all the time”.

The four items used to survey the ability to study were also taken from the DEGS questionnaire [27]. The items originally used to assess the ability to work can also be applied to students. For example, respondents were asked to rate the statement “I could not work as long as usual” on a five-point Likert scale (1 = “never” to 5 = “always”).

A validated German version of the perceived stress scale was used to measure the experience of stress (Cronbach’s alpha = 0.84) [29]. The subscale Helplessness with its six items was used. An example item was “In the last month, how often have you been upset because of something that happened unexpectedly?” The corresponding response scale ranged from 1 = “never” to 5 = “very often”.

The DRAMMA model formed the basis for the item to assess detachment. Kujanpää et al. [30] described the six dimensions (detachment, relaxation, autonomy, mastery, meaning, affiliation) with three items each. In the present study, only one item per dimension was elicited, namely, the one with the highest discriminating power on the specific dimension. In addition, “work” was replaced by “studies”. The item for the dimension detachment was “During my last evenings off, I distance myself from my studies” (1 = “I do not agree at all” to 5 = “I fully agree”).

The variables of social support from lecturers and fellow students were taken from the Berlin Demands Resources Inventory for Students [31]. Here, support from lecturers was elicited by five items (Cronbach’s alpha = 0.86), for example, “I receive help and support from lecturers whose courses I attend” and support from fellow students by four items (Cronbach’s alpha = 0.83), for example, “When I want to discuss course-related questions, I find fellow students who take time and listen well”. The response categories ranged from 1 = “never” to 6 = “always”.

#### 2.2.3. Data Analysis

The statistical analysis of the data was performed with IBM SPSS Statistics Version 27. Pearson’s product-moment correlation coefficient was used to calculate the relationships between presenteeism and the assumed outcome variables (Hypotheses 1a–1c) as well as between presenteeism and the variables characteristic of online teaching (Hypotheses 2a–2c). To improve clarity, two separate correlation tables were created. The internal consistency (Cronbach’s alpha) was satisfactory to mostly good for all scales. The items assessing ability to study were recoded so that a high value stood for a good ability to study.

In addition to the correlations, a hierarchical linear regression analysis was conducted to test how much variance in presenteeism was explained by the independent variables of stress experience, detachment, and support. In the first step, control variables should be included. In this case, the general state of health was included on a theoretical basis, since impaired health is not only a long-term consequence of presenteeism, but in a certain way also a prerequisite for presenteeism since healthy people cannot exhibit presenteeism. This can also be seen in the definitions that very often contain the description “worked despite illness/despite health impairments” [7,12,32,33]. In the next steps, the independent variables were included one after the other, beginning with the stress experience, since relationships between presenteeism and this construct have already been studied several times in the work context, and then detachment and the two support variables. Bootstrapping was also used to obtain more robust estimates. The confidence interval was set at 95% based on 10,000 bootstrapping samples.

### 2.3. Results

Table 2 gives an overview of the prevalence of presenteeism. The proportions of those who at least once in the preceding 12 months had persevered in their studies even though they felt really sick (61.4%) and who at least once deferred recovery until the completion of an important task (52.5%) are noteworthy. The remaining two items were rated much lower; 17% reported that they had pursued their studies against medical advice at least once and 10.8% had been prescribed medication at least once so as to be able to continue studying.

Table 3 shows the descriptive statistics as well as the reliability of the constructs presenteeism, general state of health, well-being, ability to study, the experience of stress, detachment, support from lecturers, and support from fellow students. In the case of support from lecturers, it should be noted that those students were excluded from the analysis who reported that they had not needed support from lecturers, which explains the low *N*.

Hypotheses 1a to 1c concerned the correlations between presenteeism and health status, well-being, and ability to study. The results are shown in Table 4. Presenteeism was statistically significantly negatively related to general health (*r* = −0.22, *p* ≤ 0.001), well-being (*r* = −0.20, *p* ≤ 0.001), and ability to study (*r* = −0.27, *p* ≤ 0.001). Consequently, Hypotheses 1a to 1c could be confirmed. Furthermore, Table 4 shows that general health, well-being, and ability to study were statistically significantly positively correlated with each other.

The correlations of presenteeism with the variables representing the characteristics during remote studying are presented in Table 5. Consistent with the hypotheses, statistically significant negative relationships appear between presenteeism and detachment (*r* = −0.27, *p* ≤ 0.001), support from lecturers (*r* = −0.12, *p* ≤ 0.01), and support from fellow students (*r* = −0.06, *p* ≤ 0.05). A statistically significant positive relationship was found between presenteeism and experiencing stress (*r* = 0.30, *p* ≤ 0.001). Hypotheses 2a to 2c could thus also be confirmed. It is moreover shown that the support scales, as well as detachment, were statistically significantly positively correlated with each other, and experience of stress was statistically significantly negatively correlated with the other variables.

A hierarchical linear regression analysis was conducted to determine how much variance in presenteeism was explained by the independent variables of experience of stress, detachment, and support. Table 6 shows the F-values and the regression coefficients including standard errors as well as the standardized regression coefficients in the different steps of the regression analysis. The results of the bootstrapping are also reported. This indicates significant results if the zero is not contained in the confidence interval. The penultimate column reports the explained variance (adjusted R^2^), i.e., how much variance in presenteeism is explained by each model, and the last column reports the change in this.

All models explain the variance of the variable presenteeism (Model 1: *F* = 28.54, *df* = 1, *p* ≤ 0.001, *R*^2^ = 0.05; Model 2: *F* = 23.18, *df* = 2, *p* ≤ 0.001, *R*^2^ = 0.08; Model 3: *F* = 20.74, *df* = 3, *p* ≤ 0.001, *R*^2^ = 0.10; Model 4: *F* = 13.54, *df* = 5, *p* ≤ 0.001, *R*^2^ = 0.11). However, Model 4 makes no further significant contribution to explaining variance. Thus, the inclusion of the two support variables does not explain any additional variance in presenteeism (∆*R*^2^ = 0.01, *p* = 0.08). The positive regression coefficient for support from fellow students, in contrast to the previous negative correlation, is striking. This can be explained by the obvious common variance of the two support variables (see discussion for more detail). In addition, both values fluctuate around zero, which indicates a low relevance of the relationship.

### 2.4. Discussion

The study demonstrates the prevalence of presenteeism at the university studied here. The result that in the preceding 12 months 61.4% of the students persevered with their studies despite feeling really sick is consistent with other student surveys [10] or is slightly below these [11]. Negative correlations were found with the constructs of general health, well-being, and ability to study, which means that high levels of presenteeism are associated with poorer health, poorer well-being, and poorer ability to study. The results of various presenteeism studies from the work context [6,7] can thus be transferred to the study context. The results obtained here are also consistent with those of a study on students defining presenteeism as impaired performance due to health problems [15].

Regarding the current trend towards increased remote studying, it was assumed that studying at home primarily increases the experience of stress, makes it more difficult to switch off, and reduces the support available from the university. As expected, there was a moderate positive relationship between the experience of stress and presenteeism. Along with this, it could also be shown in the work context that a high experiencing of stress leads to increased presenteeism [21]. Furthermore, a moderate negative relationship was shown between detachment and presenteeism. This result is also in line with the literature, as a low detachment has been associated with impaired recovery, which leads to a deterioration of the state of health [24]. This, in turn, makes presenteeism more likely [25]. With regard to support from lecturers and fellow students, a negative (albeit weak) relationship with presenteeism was found in each case. This is consistent with the results for employees, where a negative relationship between organizational support and presenteeism was found [21]. In contrast, the hierarchical linear regression analysis reveals that the variance explanation of presenteeism cannot be significantly enhanced by the inclusion of the two support variables. In addition, support from fellow students yielded a positive (though not significant) regression coefficient. This can be explained by the fact that with respect to fellow students, in contrast to lecturers, the following aspect may be relevant: students feel obliged to their fellow students and do not want to burden them, which may lead to increased presenteeism. All in all, the experience of stress and the lack of detachment seem to be more closely related to presenteeism.

#### Limitations and Future Research

Despite the insights achieved to bridge the research gap in student presenteeism, some limitations of the study need to be mentioned. First, this was a cross-sectional study. Consequently, no clear conclusions about causality can be drawn. However, various longitudinal studies from the work context have been able to show, for example, the consequences of presenteeism for health, well-being, and work ability [6,7]. For future studies, it would nevertheless be advisable to collect longitudinal data to confirm the results obtained. Secondly, it can be assumed that students who are generally interested in the topic of health tended to participate in the health survey. This aspect of self-selection was counteracted by integrating competitions, etc. when advertising the survey in order to create external incentives to participate. Thirdly, the general state of health and detachment were measured with only a single item. The use of validated scales, whose quality could have been determined by calculating reliability, would have been more appropriate. However, there are also advantages in single-item measurements, such as a higher practicability due to shorter questionnaires. Furthermore, single-item measurements also represent a valid measurement approach [34].

The study provides initial indications that the variables experience of stress and detachment explain presenteeism (Model 3: *R*^2^ = 0.10). Future studies should start here and identify further significant contributory factors. In conjunction with this, the study conducted does not yet shed light on the reasons for presenteeism apart from remote studying. For this purpose, a qualitative follow-up study was conducted, which is presented below.

## 3. Qualitative Interviews

### 3.1. Theoretical Background and Research Question

After finding evidence in the quantitative study that the experience of stress and a lack of detachment are related to presenteeism, further reasons for presenteeism—also independent of digital learning—were sought. To gain a comprehensive understanding, qualitative interviews were chosen as the method of data collection. Qualitative surveys are generally rather poorly represented in presenteeism research. Miraglia and Johns [25] point out, however, that they are of relevance for understanding whether presenteeism or absenteeism is exhibited.

Precise knowledge of the reasons for presenteeism is very important for the target group of students in order for the university to be able to implement appropriate measures. For example, it must be clear whether the context or individual reasons play a role in the behavior of working despite illness.

The reasons for presenteeism have been researched very little in the student context so far. Therefore, results from the work context will be used again. Lohaus and Habermann [21] mention in their review person-related, work-related, and organizational variables that influence the decision to engage in presenteeism. Person-related variables conducive to presenteeism are, for example, perceived stress or emotional exhaustion. Unfavorable work-related variables are, for example, workload or time pressure. At the organizational level, a strict absence policy or understaffing are conducive to presenteeism [21]. A qualitative study examining employees at a university identified the following reasons for presenteeism: quantitative workload (work-related), sense of duty (person-related), and the feeling that one is still able to perform (person-related) [20]. Presenteeism seems to be exacerbated in the home office because the barriers to working while sick are lower at home [35]. On the one hand, this is due to the fact that the adjustment latitude and temporal flexibility are greater (for example, breaks can be taken when necessary). On the other hand, people often feel that they have to work at least a little, despite their illness because they are at home anyway [36].

The findings of the few existing studies on reasons for presenteeism among the target group of students are as follows: Critz et al. [37] reported differences between the various universities surveyed. However, the following aspects were rated as the most important in each case: missing critical information, lack of opportunity to make up for missed lecture time, and fear of not passing the next exam [37]. Among medical students, it was shown that students engaging sometimes or often in presenteeism had a higher propensity to wear themselves out, as well as a poorer ability to distance themselves than did students who rarely or never exhibited presenteeism [38]. Johansen [39] studied 16–19-year-old pupils whose reasons for presenteeism can be used as comparative data. He showed that extrinsic motives (important content is explained at school, fear of negative effects on grades, and school attendance requirements) play a role. Some participants also named intrinsic motives, such as maintaining a social network, consideration for classmates, or interest in the learning content [39]. Due to the limited number of studies on the reasons for student presenteeism, the following exploratory research question is formulated rather than hypotheses:

**Research Question** **1.** *What are the main reasons for engaging in presenteeism among students?*

### 3.2. Materials and Methods

#### 3.2.1. Procedure and Participants

The interviews were conducted via Zoom over a two-week time period from late November to early December 2021. Eleven interviews with an average duration of 20 min were held. All interviews were recorded for subsequent transcription. The interview recordings comprise approximately 227 min. The general prerequisite for participation in the qualitative interviews was that subjects have already studied in the past despite illness.

Regarding the sample, the following characteristics can be mentioned: The students came from three different faculties (management sciences, natural sciences, social policy) as well as different semesters (semesters one to seven) in order to obtain a result as representative as possible. The age ranged from 20 to 28 years, with an average age of 22.18 years. Nine women and two men participated.

#### 3.2.2. Interview Guideline

The interviews dealt with the topic of presenteeism among students in an all-encompassing manner. In the context of this article, however, only the results concerning the reasons are presented. For this purpose, the open question “What reasons did you have for studying despite illness?” was asked. Following this, the reasons mentioned were summarized by the interviewer as a confirmation question and the interviewer asked whether any other reasons were relevant. In order to cover various influential factors, the study conditions (“What role do study conditions play in presenteeism?”) and individual characteristics (“In your opinion, which individual characteristics lead to presenteeism?”) were then focused on.

Subsequently, ten scaling questions were used. An example statement is as follows: “I was afraid of missing important information”. The statements were rated on a scale from 1 (“does not apply at all”) to 5 (“applies fully”). The studies by Johansen [39] and Critz et al. [37] served as the basis for the formulation of the scaling questions. According to the results reported by those authors, a large part of the statements assessed as most relevant for presenteeism were also used in the present study. In addition, a study by Hägerbäumer [33] examining presenteeism among employees using scaling questions, among other things, was used as a basis. In order to investigate two areas not yet covered by the other two studies (quantitative workload and remaining capacity), statements were taken from Hägerbäumer’s study [33] and partly adapted to the university context by minor modifications.

#### 3.2.3. Data Analysis

Qualitative content analysis according to Mayring [40] was used to evaluate the open-ended questions. The analysis technique of summary, which was applied here, aims at reducing the interview material to the substantial contents. The first step was to paraphrase the interviews. The paraphrased statements were then generalized using the same level of abstraction. This was followed by a reduction through the deletion of paraphrases with the same meaning and transfer to a summarizing category system. The categories were thus derived from the material itself. The three open-ended questions on the reasons were evaluated together in this process. In the last step, a quantitative analysis of the frequencies of the categories was carried out. Mean values and standard deviations were calculated for the scaling questions.

### 3.3. Results

Table 7, Table 8 and Table 9 provide an overview of the results of the open-ended questions. The following three superordinate categories: “Study-related influential factors”, “Personality traits”, and “Personal motives” were derived.

Four subcategories were formed regarding study-related influential factors. Missing important content was named as the most frequent reason for presenteeism. Requirements of the university (e.g., attendance rules) and the quantitative workload, especially submissions and deadlines, also played a role. Fellow students whom one does not want to let down or be a burden on were also relevant.

The students interviewed enumerated various personality traits as reinforcing presenteeism behavior. Ambition was of great importance. Conscientious individuals also had a greater tendency to presenteeism. Low self-efficacy, i.e., not trusting oneself to manage one’s studies despite interruptions due to illness, also played a role.

In the last superordinate category, personal motives were summarized. The students reported that a positive attitude towards their studies, a guilty conscience towards themselves, a hoped-for distraction from their illness, and maintaining their social network could all lead to presenteeism.

The scaling questions also revealed that the fear of missing information was rated, on average, as the strongest reason for presenteeism among students (*M* = 4.27). The two statements on quantitative workload were also rated quite highly (*M* = 3.36–3.82). In addition, the personal motives of interest (*M* = 3.64) and pleasure in studying (*M* = 3.18) played a role. A new aspect was the feeling of still being capable enough (*M* = 3.45), which was not addressed in the open questions. In summary, the scaling questions yielded similar results to the open-ended questions. The exact results can be found in Table A1 (Appendix A).

### 3.4. Discussion

The qualitative study investigated the reasons for presenteeism among students. Open questions as well as scaling questions were evaluated, with comparable results. The open-ended questions revealed that the most common reason for presenteeism was not wanting to miss anything. Regarding the study-related influential factors, university requirements, quantitative workload, and consideration for fellow students were identified. Personality traits, such as a high degree of ambition, and personal motives, such as a positive attitude towards studies were also relevant. The scaling questions also revealed that students most often exhibit presenteeism because they do not want to miss anything. The reasons of quantitative workload, interest, and fun in studying, as well as the aspect of still feeling capable enough, were also rated highly. 

The main results are comparable to those of the study by Johansen [39] from the school context. Johansen identified as the most relevant reason for presenteeism that crucial material is explained at school, which can be equated with the aspect of “missing important content”. In addition, the aspects of attendance requirements, consideration for classmates, interest in learning content, and maintenance of the social network, which are relevant here, were also identified in Johansen’s study [39]. The study by Critz et al. [37] with students also showed that the relevant reasons for presenteeism were missing critical information and the associated lack of opportunity to make up for missed lectures. In contrast to the studies by Johansen [39] and Critz et al. [37], the results of the study conducted here did not feature aspects such as fear of bad grades or of not passing the next exam. Kötter et al. [38] showed the importance of personality traits. They found that in the group of students with increased presenteeism the propensity to wear oneself out was significantly higher and in the group with low presenteeism the ability to distance oneself was significantly higher. The present study also revealed personality traits (e.g., ambition) conducive to presenteeism.

#### 3.4.1. Limitations and Future Research

As described, Miraglia and Johns [25] point out that qualitative research is useful for understanding the decision-making process in favor of or against presenteeism. Consequently, the qualitative interviews conducted here were able to elicit much more individual reasons than is possible, for example, with questionnaires. Nevertheless, the limitations of the research must be conceded. In general, especially in non-anonymous interview studies, there is the problem of social desirability and the possible accompanying bias in the responses of the students interviewed. Another issue is the composition of the sample. For future studies, it would be advisable to interview even larger samples to be able to differentiate in greater detail in the evaluation, for example, according to gender, field of studies, or stage of studies. It is possible that this would reveal differences in presenteeism behavior.

Especially in personal characteristics, there are certainly even more influential factors than those highlighted here. For example, work engagement is positively and optimism negatively related to presenteeism [21]. Other interesting variables could be, for example, students’ resilience or self-regulation, whose influence on presenteeism should be quantitatively investigated in future studies. It is also possible that negative personality traits (e.g., selfishness, lack of responsibility towards others) could explain the choice to be present on courses despite illness. If presenteeism is shown in presence at the university, it is no longer only the health of the individual that is affected, but also the health of the other students. This is about the topic of solidarity. In contrast, one of the reasons given for presenteeism in the interviews was consideration for fellow students. This means that it depends on the situational conditions whether solidarity or contrary characteristics lead to presenteeism or not, which is an interesting field of research.

Beyond these individual characteristics, it can be assumed that belonging to different cultural groups has an influence, as presenteeism occurs more frequently in some cultures than in others. For example, it has been shown for employees that presenteeism is more common among Chinese employees than among British employees [41]. Further research should be conducted regarding students in order to ascertain whether cultural differences can also be uncovered for this target group.

Another issue that should be investigated in the future regarding presenteeism among students is the financial impact of presenteeism on the university and on the students. For example, costs are incurred when courses have to be canceled due to the contagion of lecturers by a student. Other examples regarding students are a negative impact on grades due to presenteeism, which also shows up on the report card or their individual health care costs.

An interesting field of research based on the overall results is the question of possible countermeasures that students would welcome. In this context, one would ideally survey the presenteeism behavior of students once again after the introduction of the measures to ascertain their effectiveness.

#### 3.4.2. Practical Implications

The study showed that presenteeism is also prevalent in the student context and has negative effects on health, well-being, and the ability to study. Countermeasures are therefore recommended. As a first step, students should be made aware of the negative effects of presenteeism. This is possible, for example, in a short presentation on the topic of health as part of the first-semester orientation. Information material is also useful. In addition, the establishment of student health management, in general, makes sense. Through regular participation in health courses or workshops, in the best case, the health status of the students improves and the likelihood of any presenteeism is reduced [33]. Regarding the reasons for presenteeism, not wanting to miss anything was identified as the main reason. Against this background, students should be advised to look for a fixed learning partner whose transcripts they can rely on in case of illness and stay at home with a clear conscience. Alternatively, it is a good idea to record the lectures or make the materials available online. The supplementary use of digital, asynchronous learning materials is thus an important achievement of the Corona era that should be maintained. Furthermore, lecturers themselves should not give their lectures when ill in order not to be a negative role model [20]. The study also made clear that the effects associated with studying at home (especially more stress and detachment) correlate with presenteeism. This highlights the relevance of contact teaching in terms of students’ presenteeism behavior. It should not be completely replaced by online teaching but complemented in a meaningful way.

Independent of the points above, the question arises as to whether the phenomenon of presenteeism may be a direct result of unrealistic expectations on the part of the education system. The university has the responsibility to control this phenomenon and to regulate it through the measures proposed instead of encouraging it. Moreover, it should be mentioned that consideration for the group of students as a whole is of importance. As already noted in the introduction, today’s students are tomorrow’s workers [1]. Due to demographic change, there is a decline in the working population and thus a shortage of skilled workers [42]. It is therefore particularly important that the existing workforce should remain fit for work in the long term, which is only possible with a healthy lifestyle [1]. This issue should also be kept in mind against the background of presenteeism.

## 4. Conclusions

In summary, it can be stated that the study makes an important contribution to bridging the research gap in “presenteeism in the student context”. The quantitative student survey showed that the negative relationships with the state of health, well-being, and the ability to work or, in this case, to study, familiar from the work context, also apply to students. A special focus was also placed on digital learning, which has been intensified by the Corona pandemic and the effects of this on the presenteeism behavior of students. In particular, heightened experience of stress and impaired ability to switch off were identified as factors influencing presenteeism. The qualitative interviews evaluated with the help of qualitative content analysis according to Mayring provided a comprehensive picture of the reasons for presenteeism. Not wanting to miss anything was rated as most relevant. University requirements (e.g., attendance regulations), the quantitative workload, and consideration for fellow students also played a role. In addition to these study-related influential factors, the categories of personality traits (e.g., highly developed ambition) and personal motives (e.g., a positive attitude towards studies) also emerged. In light of these results, various practical implications could be derived by addressing the reasons listed and thus minimizing the negative effects of presenteeism on health, well-being, and the ability to study.

## Figures and Tables

**Table 1:** Representativeness of the participants.

Characteristic	Percentage in the Student Survey (*N* = 1036)	Percentage in the Population of the University (*N* = 9256)
Gender		
Female	61%	40%
Male	39%	60%
Age in years		
17–24	72%	61%
25–30	22%	27%
Over 30	6%	12%
Degree pursued		
Bachelor	83%	86%
Master’s	17%	14%
Semester of study *		
First or second	25%	24%
Third or later	75%	76%

* The semester of study characteristic only refers to bachelor’s students.

**Table 2:** Prevalence of presenteeism.

Characteristic	Not Once	Once	Twice or More
Studying while sick	38.6%	28.1%	33.3%
Recovery deferred until the completion of an important task	47.5%	27.8%	24.7%
Studying against medical advice	82.9%	7.9%	9.1%
Were prescribed medicine to be able to continue studying	89.2%	6.3%	4.5%

*N* = 1014–1023.

**Table 3:** Descriptive statistics and reliability of the scales.

Scale	Number of Items	Scaling	*M*	*SD*	Min	Max	Cronbach’s Alpha
Presenteeism	4	1–3	1.54	0.53	1.00	3.00	0.73
General state of health	1	1–5	3.25	0.78	1.00	5.00	-
Well-being	5	1–6	3.39	0.93	1.00	6.00	0.82
Ability to study	4	1–5	3.32	0.92	1.00	5.00	0.84
Experience of stress	6	1–5	3.11	0.86	1.00	5.00	0.87
Detachment	1	1–5	2.98	1.28	1.00	5.00	-
Support from lecturers	5	1–6	3.71	1.11	1.00	6.00	0.89
Support from fellow students	4	1–6	3.93	1.24	1.00	6.00	0.84

*N* = 539–1036, *M* = mean, *SD* = standard deviation, *Min* = minimum, *Max* = maximum.

**Table 4:** Intercorrelations of the variables (health status, well-being, and ability to study).

		1	2	3	4
1	Presenteeism	1			
2	General state of health	−0.22 ***	1		
3	Well-being	−0.20 ***	0.53 ***	1	
4	Ability to study	−0.27 ***	0.41 ***	0.50 ***	1

*N* = 1026–1036, *** *p* ≤ 0.001.

**Table 5:** Intercorrelations of the variables (characteristics of remote studying).

		1	2	3	4	5
1	Presenteeism	1				
2	Experience of stress	0.30 ***	1			
3	Detachment	−0.27 ***	−0.37 ***	1		
4	Support from lecturers	−0.12 **	−0.15 ***	0.10 *	1	
5	Support from fellow students	−0.06 *	−0.21 ***	0.11 ***	0.31 ***	1

*N* = 539–1036, *** *p* ≤ 0.001, ** *p* ≤ 0.01, * *p* ≤ 0.05.

**Table 6:** Hierarchical linear regression analysis.

	*F*	*B*	*SE B*	β	Bootstrapping		*R* ^2^	∆*R*^2^
					95% BC CI			
					Lower	Upper		
Model 1	28.54 ***						0.05	0.05 ***
Constant		2.11	0.09		1.93	2.30		
General state of health		−0.15	0.03	−0.23 ***	−0.21	−0.10		
Model 2	23.18 ***						0.08	0.03 ***
Constant		1.54	0.17		1.22	1.87		
General state of health		−0.10	0.03	−0.15 **	−0.16	−0.03		
Experience of stress		0.13	0.03	0.19 ***	0.06	0.19		
Model 3	20.74 ***						0.10	0.03 ***
Constant		1.81	0.18		1.45	2.17		
General state of health		−0.09	0.03	−0.13 **	−0.15	−0.02		
Experience of stress		0.10	0.03	0.15 **	0.03	0.16		
Detachment		−0.07	0.02	−0.17 ***	−0.11	−0.04		
Model 4	13.54 ***						0.11	0.01
Constant		1.85	0.20		1.44	2.27		
General state of health		−0.09	0.03	−0.14 **	−0.16	−0.03		
Experience of stress		0.09	0.03	0.14 **	0.03	0.16		
Detachment		−0.07	0.02	−0.16 ***	−0.11	−0.03		
Support from lecturers		−0.04	0.02	−0.08	−0.08	−0.00		
Support from fellow students		0.03	0.02	0.07	−0.00	0.07		

*N* = 539–1036, *** *p* ≤ 0.001, ** *p* ≤ 0.01. *B* = regression coefficient, *SE B* = standard error, β = standardized regression coefficient, BC CI = bias-corrected confidence interval, *R*^2^ = explained variance (adjusted), ∆*R*^2^ = change in *R*^2^.

**Table 7:** Results of the open-ended questions on the reasons for presenteeism (study-related influential factors).

Study-Related Influential Factors	
Categories	Entries
Missing important content	24 (9)
Fear of missing out	10
Lack of confidence in fellow students’ notes	7
Fear of losing the connection	4
Lack of opportunity to catch up on content	3
Requirements of the university	10 (7)
Attendance regulations	6
Examination phase	3
Fixed dates	1
Quantitative workload	5 (5)
Submissions and deadlines	5
Consideration for fellow students	5 (4)
Do not abandon fellow students	3
Do not be a burden on fellow students	2

The numbers in parentheses indicate how many of the respondents referred to the particular upper category.

**Table 8:** Results of the open-ended questions on the reasons for presenteeism (personality traits).

Personality Traits 19 (11)	
Categories	Entries
Ambition	8
Conscientiousness	3
Low Self-Efficacy	3
Neuroticism	2
Perfectionism	2
Extraversion	1

The numbers in parentheses indicate how many of the respondents referred to the particular upper category.

**Table 9:** Results of the open-ended questions on the reasons for presenteeism (personal motives).

Personal motives 13 (10)	
Categories	Entries
Positive attitude towards studies	6
Guilty conscience	3
Distraction from the illness	2
Maintenance of social network	2

The numbers in parentheses indicate how many of the respondents referred to the particular upper category.

## Data Availability

The data presented in this study are available on request from the corresponding author. The data are not publicly available because they were collected in a public institution (university).

## Appendix A

**Table A1:** Results of the scaling questions on the reasons for presenteeism.

Reason	*M*	*SD*	Min	Max	Assigned Category
I was afraid of missing important information.	4.27	1.42	1	5	Missing important content
I had too much to do.	3.82	1.08	2	5	Quantitative workload
I have a great interest in the contents from my studies.	3.64	0.67	3	5	Personal motives
I still felt capable enough.	3.45	0.93	2	5	Capability
There were urgent submissions and deadlines.	3.36	1.63	1	5	Quantitative workload
I enjoy my studies.	3.18	1.08	2	5	Personal motives
I did not want to burden my fellow students (e.g., in group work).	3.09	1.30	1	5	Consideration for fellow students
There was no way to make up for missed lectures.	2.82	1.08	1	4	Missing important content
I wanted to maintain my social network.	2.45	1.13	1	4	Personal motives
Attendance at lectures is compulsory.	2.27	1.68	1	5	Requirements of the university

*N* = 11, *M* = mean, *SD* = standard deviation, *Min* = minimum, *Max* = maximum. Five-point Likert scale: 1 = does not apply at all, 2 = applies a little, 3 = applies moderately, 4 = applies mostly, 5 = applies fully. To show similarities and differences to the results of the open-ended questions, the categories formed in the open-ended questions were also assigned here.
